# A preconception to late pregnancy community cohort study for reference values of serum thyrotropin and total thyroxine

**DOI:** 10.3389/fendo.2026.1757357

**Published:** 2026-07-23

**Authors:** Ashwin Valliyot, P. K. Jabbar, Sreejith U. S. Babu, Jayakumari Chellamma, Abilash Nair, Madan Godbole, Bharati Kulkarni, Ravleen Kaur Bakshi, Archana Mohan Preetha, Karthik Vijayakumar

**Affiliations:** 1 Government Medical College, Thiruvananthapuram, Thiruvananthapuram, India; 2 KLE Academy of Higher Education and Research Deemed to be University, Belagavi, India; 3 Indian Council of Medical Research, New Delhi, India

**Keywords:** anti-TPO antibody, hypothyroidism, total T4, trimester specific reference interval, TSH

## Abstract

**Introduction:**

Population-based preconception cohort studies to establish reference ranges for thyroid function tests during pregnancy are scarce. This study was done to establish the reference values for thyrotropin (TSH) and total thyroxine (T4) during the first and late-second trimesters in preconceptionally euthyroid, thyroid autoantibody-negative, community-dwelling pregnant women in a South-Indian district.

**Methods:**

Newly married nulliparous women aged 18–35 years from were recruited preconceptionally using multistage cluster sampling. Baseline pre-conceptional thyroid function, including TSH, Total T4, and anti-Thyroid peroxidase(TPO) antibodies, was assessed. Women who were euthyroid and negative for TPO antibodies were included and followed throughout pregnancy, with repeat thyroid function tests during the first and second trimesters.

**Results:**

Of the 1507 women enrolled, 482 pregnancies were identified during the 18-month follow up period. Among these, 182 met the inclusion criteria, with 125 assessed in the first trimester and 120 in the second trimester. The first-trimester TSH reference range was found to be 0.1–3.9 µIU/mL, and the second trimester range was 0.7–4.6 µIU/mL. Total T4 reference ranges were 5.2–17.0 µg/dL and 7.6–14.7 µg/dL, respectively. In the longitudinally studied women, TSH decreased from 2.3 ± 1.0 µIU/mL pre-pregnancy to 1.6 ± 1.0 µIU/mL in the first trimester, then rose marginally to 2.0 ± 0.7 µIU/mL in the second trimester. First-trimester TSH levels correlated positively with pre-pregnancy TSH and with abdominal circumference in these anti-TPO negative women (p=0.01).

**Conclusion:**

This study establishes community-based trimester-specific reference intervals for TSH and Total T4 in preconceptionally euthyroid, TPO antibody negative community dwelling Indian women.

## Background

Hypothyroidism during pregnancy, is associated with poor outcomes for both the mother and the fetus (1). Adverse maternal outcomes include spontaneous abortion, pre-eclampsia, gestational hypertension, and preterm delivery, while fetal risks include lower Intelligence Quotient (IQ) and neurodevelopmental delays (2). An inverted U-shaped relationship was observed between maternal TSH levels and offspring total grey matter volume, with the strongest impact around eight weeks of gestation (3). Children of hypothyroid women, especially those untreated during pregnancy, have lower performance on tests related to intelligence, attention, language, reading ability, school performance, and visual-motor skills.

(4). Hence diagnosis of thyroid disorders during pregnancy are of utmost importance which requires population, assay and trimester-specific TSH and total or free thyroxine (T4) reference intervals. Established reference ranges are important to guide diagnostic algorithms, treatment strategies in defined population. The American Thyroid Association (ATA) guidelines, in their most recent 2017 recommendations, advocate using population- and trimester-specific TSH reference intervals calculated in a population with no known thyroid disease, optimal iodine status, and negative anti-thyroid peroxidase antibody (TPOAb) status (5). However, existing trimester-specific thyroid hormone reference intervals cannot be generalized due to variations in ethnicity, maternal iodine status, and laboratory assay methods. Specifically, there is no population based study published from India to determine the trimester specific reference range of thyroid function test during pregnancy.

This study aims to establish trimester-specific reference values for TSH levels and Total T4 in the first and second trimesters in preconceptionally euthyroid, healthy, thyroid autoantibody-negative community-dwelling pregnant women in Thiruvananthapuram, Kerala.

## Methodology

A community-based prospective observational study was done using multistage sampling in the Thiruvananthapuram district of South India.Sample size was calculated using the formula n = Z² × SD²/d², where Z = 1.96 (95% confidence), SD is the standard deviation, and d (precision) was taken as 10% of the mean TSH value. Based on trimester-specific data from Mankar et al. (mean ± SD TSH: first trimester 1.65 ± 0.85, second trimester 2.59 ± 1.09, third trimester 2.77 ± 1.19 mIU/L), the calculated sample sizes were 102, 69, and 63 respectively.

The largest sample size (n = 102) was chosen to ensure adequate power. Considering a 10% attrition rate, the final sample size was increased to 112 participants per trimester (6). The study was done over a period of one year starting April 2023, after obtaining approval from Institutional Human Ethics Committee of Government medical college Thiruvananthapuram (Approval number:06/14/2023/MCT).

**Figure 1:**
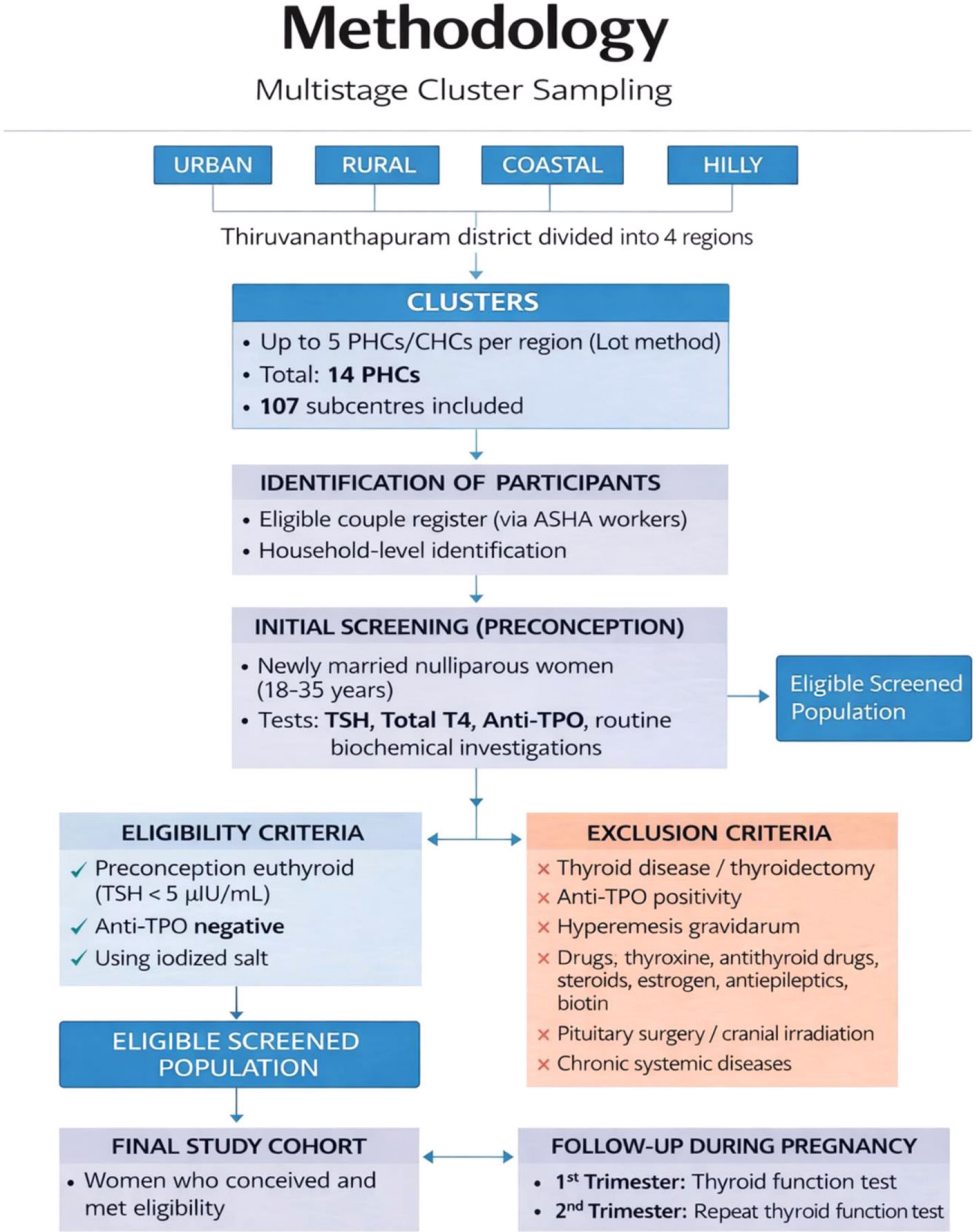
Methodology -multistage sampling.

Newly married nulliparous women aged 18–35 years from four different geographic areas were identified for the study. Preconceptional thyroid function status, including TSH, Total T4, and Anti-TPO antibodies, was assessed in all participants. Those who became pregnant were included in this study if they were preconceptionally euthyroid (TSH <5 µIU/mL) and negative for thyroid autoantibodies and consuming iodized salt. These women were followed up during the first and second trimesters with repeat thyroid function tests once in each trimester. Exclusion criteria included a history of thyroid diseases or thyroidectomy, positivity for anti-thyroid peroxidase antibodies, hyperemesis gravidarum in the first trimester, current treatment with thyroxine, antithyroid medications, steroids, or estrogen, a history of pituitary surgery, cranial irradiation, use of antiepileptic medications or biotin, and chronic systemic diseases such as chronic kidney disease, chronic liver disease, or autoimmune rheumatological disorders.Multistage cluster sampling was employed to ensure representative sample of entire study population in district, In the first stage, Thiruvananthapuram district was divided into 4 socio- geographic regions as urban, rural coastal and hilly. For the next stage,within each region, up to five Primary Health centers or community health centers were randomly selected using lot method. All family welfare sub-centers within the above PHCs or CHCs were included in the study. The eligible couple register maintained at the sub-centers with the help of Accredited social health activist (ASHA) was used to find a household of prospective participants. A total of 14 primary health centres and 107 subcentres were included in the study (**Figure 1**). Dedicated study staff trained to conduct the survey and blood sampling conducted the initial participant recruitment.

After getting written informed consent, in the preconceptional period a detailed history was taken including sociodemographic details, symptoms, family history of thyroid disease, or any treatment and was entered in a semi-structured proforma. Clinical examination including anthropometric measurements, pulse rate, blood pressure, height, weight, abdominal circumference, body mass index and examination for goitre was done. Height measurements were taken using a Seca mobile stadiometer (Germany). Weight measurements were obtained using a Seca weighing machine (Germany). Abdominal circumference was measured using a Seca measuring tape (Germany). An average of 3 seated blood pressure measurements 3 minutes apart after 5 minutes of rest using Omron HEM 7120 (Kyoto, Japan) were done. Five ml of blood was drawn under aseptic precautions before 10 am for measuring thyrotropin (TSH), thyroxine (Total T4) and anti-thyroid peroxidase antibody (ATPO). The collected blood sample was transported to a central laboratory maintaining cold chain at 2–8 degrees celsius as required. Additionally, samples were collected for Fasting plasma glucose, 2-hour Postprandial glucose, alanine aminotransferase(ALT), aspartate amino transferase(AST), glycosylated hemoglobin(HbA1c), fasting insulin, total cholesterol, fasting triglycerides, high density lipoprotein(HDL) and low density lipoprotein(LDL).

After pregnancy was detected, samples for TSH and Total T4 were taken during 1^st^ trimester (10–12 weeks) and during 2^nd^ trimester (24–28 weeks). In the first trimester, pregnant women with TSH levels greater than 4 μIU/mL were included in the first trimester analysis. However, these individuals were excluded from the follow-up study and referred to the Endocrinology outpatient service for considering treatment. In the second trimester, participants with TSH levels exceeding 4 μIU/mL were included in the final analysis and referred to the outpatient clinic for treatment consideration.Serum TSH, Total T4 and TPO antibodies was measured on the Roche e411 immunoassay analyser which employs the electrochemiluminescence immunoassay (ECLIA) sandwich principle for TSH and ATPO and competitive immunoassay for Total T4. ‘Elecsys TSH, Elecsys T4, Elecsys Anti-TPO kit were used which was non-competitive two site assay. The coefficient of variation was 8.7% at lower values and 1.8% near TSH values of 10 µIU/mL. Similarly – T4 at lower range 2.2% and higher range 4.3%. For anti TPO, coefficient of variation at 15IU/L was 7% and 4.2% at mean concentration of 269 IU/mL. Data were analyzed using IBM SPSS Statistics v27 (2022). Continuous variables are expressed as mean ± SD or medians with interquartile ranges (IQR) based on their distribution, while categorical variables are presented as frequencies and percentages. Trimester-specific reference intervals for TSH and Total T4 were established via non-parametric methods at the 2.5th and 97.5th percentiles. Differences across timepoints for the longitudinal cohort were evaluated using paired t-tests. Differences between independent groups were analyzed using one-way ANOVA for continuous variables and Chi-square or Fisher’s exact tests for categorical data. Non-parametric bivariate correlations were evaluated using Spearman’s rank correlation coefficients. To identify independent predictors of a significant TSH decline (upper 10th percentile), relevant baseline clinical and biochemical variables were entered into a multivariable logistic regression model. Statistical significance was set at a two-tailed <0.05.

## Results

**Figure 2:**
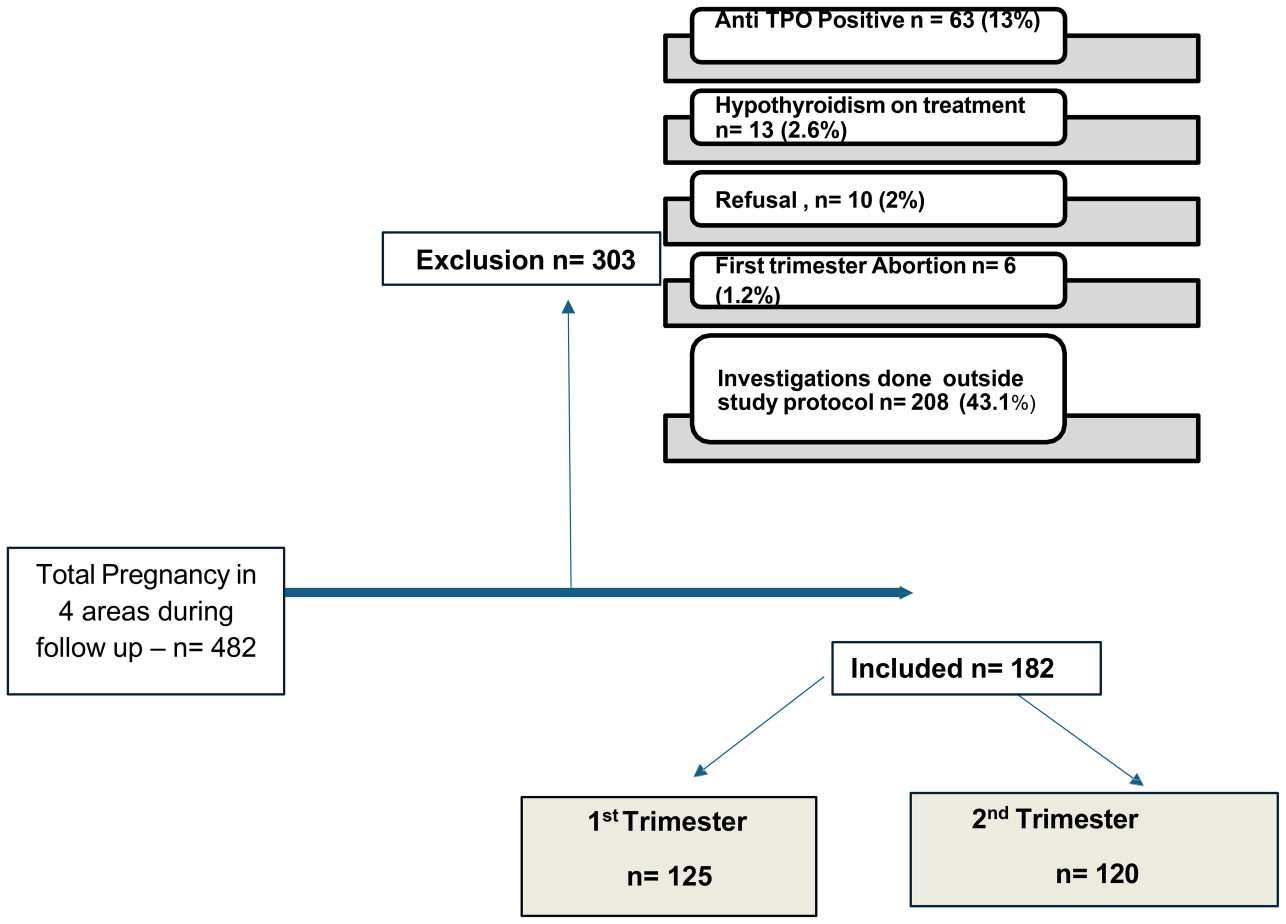
Participant flow.

The study initially included 482 pregnancies from four different regions in Thiruvananthapuram district 182 women met the inclusion criteria (**Figure 2**). Among these, 125 participants were in first trimester and 120 were in the second trimester. Sixty-three women had complete follow-up with samples collected during the pre-pregnancy period and both trimesters.

Among four different geographic areas in Thiruvananthapuram district, the coastal region contributed the largest proportion of participants (n=64;, 35.2%), followed by the urban area (n= 56; 30.8%), hilly (n=34; 18.7%), and rural (n=28; 15.4%). The socioeconomic distribution showed that 40.5% (74 participants) belonged to the lower middle class, 36.3% (64 participants) to the upper lower class, and 22.5% (41 participants) to the upper middle class, with the upper and lower socioeconomic classes each accounting for 0.5% of the cohort.

The baseline demographic profile revealed a mean age of 23.8 ± 3.3 years and the mean BMI of 23.2 ± 4.4 kg/m². The mean abdominal circumference was 78.5 ± 9.7 cm. Laboratory results showed a mean fasting plasma glucose level of 84.4 ± 9.2 mg/dl and a postprandial plasma glucose level of 100.1 ± 30.2 mg/dl. LDL cholesterol levels had a mean of 121.1 ± 33.4 mg/dl. HDL cholesterol levels averaged 51.2 ± 11.6 mg/dl as described in **Table 1** Mean pre-pregnancy TSH was 2.45 ± 1.1 µIU/mL, mean total T4 was 8.8 ± 1.8 µg/dL, and mean anti-TPO was 13.2 ± 4.3 IU/mL. TSH did not differ significantly across age groups, BMI categories, or geographic areas.

**Table 1:** Baseline demographic, clinical and biochemical characteristics of study population.

Parameter	Mean ± standard deviation
Age (Years)	23.8 ± 3.3
Height(cm)	156.6 ± 5.5
Weight (kg)	57.1 ± 11.5
BMI(kg/m2)	23.2 ± 4.4
Abdominal Circumference(cm)	78.5 ± 9.7
Pulse rate (bpm)	82.4 ± 12.0
Systolic BP (mm Hg)	107.25 ± 9.4
Diastolic BP (mm Hg)	71.41 ± 7.7
Fasting Plasma Glucose (mg/dL)	84.4 **±** 9.2
Postprandial Plasma Glucose (mg/dL)	100.1 ± 30.2
AST(IU/L)	25.3 ± 13.0
ALT(IU/L)	22.5 ± 21.6
HbA1C (%)	5.2 ± 0.3
Fasting Insulin Levels mIU/L	8.8 (5.2-13.1)
Total Cholesterol (mg/dl)	180 (158 -203.5)
Triglyceride (mg/dl)	80 (61-100)
LDL (mg/dl)	111.5 (100-135.8)
HDL (mg/dl)	48.9 (41.6- 56.7)

TSH levels for the 97.5th centile during the first and second trimesters showed a first-trimester mean of 1.69 ± 1.0 µIU/mL (2.5th centile: 0.10 µIU/mL, 97.5th centile: 3.9 µIU/mL(95% CI: 3.59–4.39) and median of 1.5 µIU/mL (IQR 0.9-2.47)and a second trimester mean of 2.13 ± 0.8 µIU/mL (2.5th centile: 0.7 µIU/mL, 97.5th centile: 4.6 µIU/mL(95% CI: 4.15–5.05),with median of 2 µIU/mL(IQR 1.6-2.5) (**Table 2**). Total T4 levels showed a first trimester mean of 11.0 ± 2.5 µg/dl (2.5th centile: 5.2 µg/dl, 50th centile: 10.8 µg/dl, 97.5th centile: 17.0 µg/dl) and a second trimester mean of 11.4 ± 1.8 µg/dl (2.5th centile: 7.6 µg/dl, 50th centile: 11.2 µg/dl, 97.5th centile: 14.7 µg/dl).

**Table 2:** Distribution of tsh values across trimesters with percentile-based reference limits.

TSH (µIU/mL)	Mean ± standard deviation	2.5^th^ centile	50^th^ centile	97.5^th^ centile
1^st^ Trimester	1.69 ± 1.0	0.10	1.5	3.9
2^nd^ Trimester	2.13 ± 0.8	0.7	2.0	4.6

**Figure 3:**
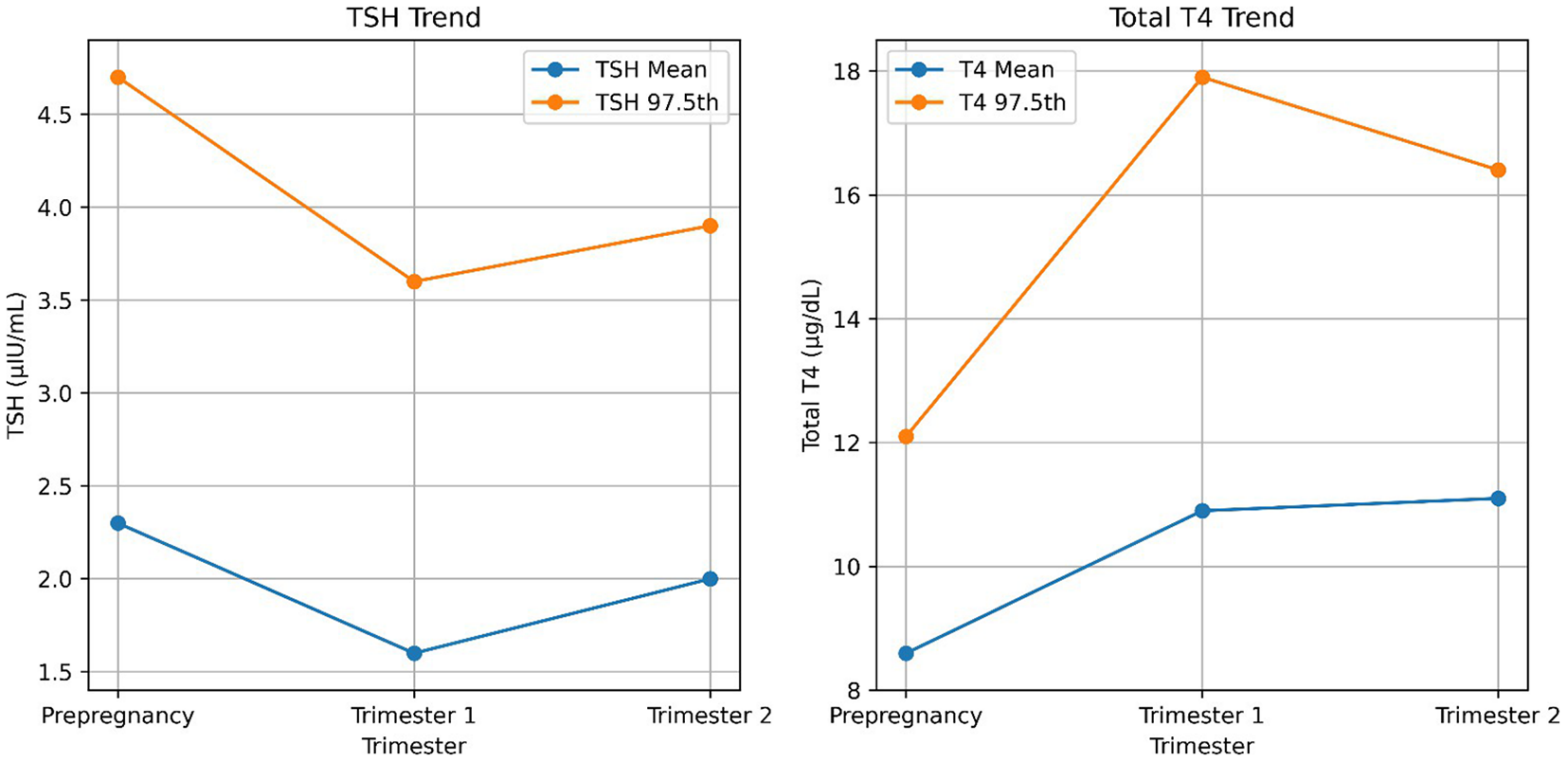
TSH and total T4 from prepregnancy to second trimester.

**Figure 3** depicts the trend of TSH (in µIU/mL) and total T4 (in µg/dl) levels in 63 patients from pre-pregnancy through the first trimester to the second trimester. Mean TSH levels decreased significantly from a pre-pregnancy value of 2.3 ± 1.0 µIU/mL to 1.6 ± 1.0 µIU/mL in the first trimester (P<0.01)) and then slightly increased to 2.0 ± 0.7 µIU/mL (P = 0.02) during the second trimester. The 97.5th percentile for TSH shows a decreasing trend from 4.7 µIU/mL pre-pregnancy to 3.6 µIU/mL in the first trimester, followed by an increase to 3.9 µIU/mL in the second trimester.

For T4 levels, the mean rises from 8.6 µg/dl pre-pregnancy to 10.9 µg/dl (P<0.01) in the first trimester and then remains stable at 11.1 µg/dL in the second trimester(P = 0.55). The 97.5th percentile for T4 shows a increase from 12.1 µg/dL pre-pregnancy to 17.9 µg/dL in the first trimester, thereby decreasing to 16.4 µg/dL in the second trimester.

According to the previous 2011 ATA guideline, with a fixed cutoff of TSH>2.5 µIU/mL for the diagnosis of hypothyroidism, 24.8% (31 out of 125) participants would have received thyroxine treatment, and with a cutoff of TSH> 3 µIU/mL for the second trimester, 15.8%(19 of 120) participants might have received thyroxine treatment (**Table 3**).

**Table 3:** Diagnosis of hypothyroidism based on previous ata (2011) tsh cut-offs.

Trimester	Participants with raised TSH	Percentage
First Trimester (>2.5 µIU/mL)	31/125	24.8%
Second Trimester (>3.0 µIU/mL)	19/120	15.8%

The determinants of first-trimester TSH were pre-pregnancy TSH and abdominal circumference. Those with higher prepregnancy TSH values had higher first trimester TSH values.(Spearman coefficient 0.4, P<0.01)Similarly those with higher prepregnancy abdominal circumference had higher TSH in first trimester (Spearman coefficient 0.1, P = 0.01).

On examining the correlation between pre-pregnancy TSH levels and the subsequent decline in TSH after conception and during the first trimester, participants in the highest decile of TSH reduction (upper 10^th^ centile) had significantly higher pre-pregnancy TSH levels (4.5 ± 0.9 µIU/mL) compared to with the smallest reduction (2.2 ± 0.9 µIU/mL) (P<0.05).

Women in the highest decile of TSH reduction (upper 10^th^ centile) had a lower pre-pregnancy total cholesterol (185 ± 38.4 vs 162.2 ± 34.4 mg/dl) and HDL (50.4 ± 11.6 vs 41.9 ± 8.9 mg/dl) compared with the rest of the cohort. On logistic regression analysis, pre-pregnancy TSH and pre-pregnancy total cholesterol were found to have an independent association with a greater fall in TSH levels. None of the other factors including total T4, age, height, weight, BMI, abdominal circumference, pulse rate, systolic and diastolic blood pressure, fasting plasma glucose, post prandial plasma glucose, SGOT, SGPT, triglyceride, LDL, HbA1C, fasting insulin showed a significant association with decline in TSH levels.

## Discussion

This prospective cohort study was done to address the dearth of community based studies regarding normal values of thyroid function in Indian pregnant women. Through a community based, multistage sampling strategy starting recruitment in the preconceptional period,current study has established the trimester specific reference interval for TSH and Total T4 in first and late second trimester of pregnancy in healthy Indian women for the first time. To the best of our knowledge there are no previous studies who report on preconceptional thyroid function tests with repeat thyroid hormone estimations during pregnancy giving us a unique opportunity to study the changes in thyroid function around conception. Previous guidelines and studies report on the trimester specific thyroid function which have been compared to the current study below. Recommendations from the American Thyroid Association (2011), the Endocrine Society (2012), and the European Thyroid Association (2014) had advised using globally applicable trimester-specific reference ranges for TSH (eg 2.5 µIU/mL in first trimester). These recommendations were based on earlier studies reporting TSH cutoffs detailed in **Table 4**. For example, Soldin et al. (2006) in Sweden reported first-trimester TSH reference intervals of 0.03–2.99 µIU/mL and second-trimester intervals of 0.35–2.95 µIU/mL (7). Similarly, Bocos-Terraz et al. (2009) in Spain reported upper limits of approximately 2.6 µIU/mL in both trimesters (8), while Stricker et al. (2007) in Switzerland also demonstrated comparably lower upper reference limits (9). Haddow et al. (2004) reported slightly higher upper limits, although subsequent studies suggested only modest variation in these thresholds.More recent data indicate a shift towards higher upper reference limits. Dorizzi et al. (2023) in Italy reported first-trimester reference intervals of 0.34–3.81 µIU/mL and second-trimester intervals of 0.68–4.07 µIU/mL (11). Similarly, Yanachkova et al. (2024) in Bulgaria reported upper limits approaching 2.9 µIU/mL in the first trimester and 4.2 µIU/mL in the second trimester (12). These findings align closely with our study results and highlight the relevance of the 2017 ATA recommendation to use population-based, trimester-specific reference intervals (10).

**Table 4:** TSH reference range comparison with previous studies.

Author	Setting	Assay	First trimester TSH reference range (µIU/mL)	Second trimester TSH reference range (µIU/m l)
Soldin et al (2006) (7)	Obstetric clinic	CLIA (Dade Behring Inc. RxL Dimension Clinical Chemistry Analyzer)	0.24–2.99	0.35–2.95
Bocos Terraz et al. (2009) (8)	Hospital	CLIA (ARCHITECT i2000SR, Abbott)	0.10–2.65	0.12–2.64
Stricker et al (2007) (9)	Prenatal clinic	CLIA (ARCHITECT i2000SR, Abbott)	0.09-2.83	0.20-2.79
Haddow et al (2004) (10)	Antenatal clinic	CLIA (Immulite2000)	0.08–2.73	0.39–2.70
Dorizzi et al (2023) (11)	Hospital	CLIA (Roche)	0.34 -3.81	0.68 - 4.07
Yanachkova et al (2024) (12)	Hospital	CLIA (Roche Cobas 8000)	0.49-2.91	0.73-4.22
Current study (2024)	Community, India	CLIA (Roche Cobas e411)	0.1-3.9	0.7-4.6

A hospital-based study from from north India by Marwah et al. (2008) reported TSH reference ranges of 0.6-5.0 µIU/mL and 0.4-5.7 µIU/mL for the first and second trimesters, respectively (13). Similarly, a prospective longitudinal study from north India by Sekhri et al. (2016) involving five hospitals in Delhi with 86 participants reported the widest TSH ranges of 0.09-6.65 µIU/mL in first and 0.51-6.66 µIU/mL in second trimester (14). Rajput et al. (2016) at PGI Rohtak reported TSH ranges of 0.37-3.69 µIU/mL and 0.54-4.47 µIU/mL in first and second trimester respectively (15) Studies from Eastern India also showed wide variability in reference ranges. Maji et al. from Kolkata reported included 195, 205, and 110 participants across the trimesters, with TSH ranges of 0.25-3.35 µIU/mL and 0.78-4.96 µIU/mL for the first and second trimester (16). Similar study from the same area by Pramanik et (2020) included 100 participants per trimester and estimated urinary iodide levels, confirming iodide sufficiency, with TSH ranges of 0.19-4.34 µIU/mL and 0.46-4.57 µIU/mL in 1^st^ and 2^nd^ trimester respectively (17). Jeba Singh et al. (2016) from Manipur reported the lowest TSH references, although anti thyroid peroxidase antibody levels were not assessed in the study (18).

Our findings show that the TSH levels in the Thiruvananthapuram population are higher in both the first and second trimesters compared to many previous international studies which contributed to the 2011 ATA recommendations for fixed TSH cutoff of 2.5 µIU/mL for the first trimester and 3 µIU/mL for the second.However, the study findings are more consistent with the ATA 2017 recommendations and with recent studies from Italy by Dorizzi et al. and Bulgaria by Yanachkova et al, which report higher upper limits for TSH (11, 12). Similar to these findings, Indian studies also demonstrate a wide range of TSH levels as shown in **Table 5**, highlighting the variability in thyroid function across populations, even within geographically proximal regions.

**Table 5:** TSH reference range comparison with other indian studies.

Author	Setting	Assay	First trimester TSH reference range (µIU/mL)	Second trimester TSH reference range (µIU/mL)
Marwah et al	Tertiary care	CLIA	0.6-5.0	0.4-5.7
Maji et al	Tertiary care	ELISA	0.25-3.35	0.78-4.96
Sekhri et al	Tertiary care	CLIA	0.09-6.65	0.51-6.66
Jebasingh et al	Tertiary care	CLIA	0.21-1.81	0.72-1.71
Rajput et al	Tertiary care	CLIA	0.37-3.69	0.54- 4.47
Pramanik et al	Tertiary care	CLIA	0.19-4.34	0.46-4.57
Current study	Community	CLIA	0.10-3.9	0.7-4.6

The differences in reference intervals observed in our study compared to other populations are likely multifactorial, reflecting variations in iodine status as well as genetic and ethnic influences on thyroid physiology. Previous studies has demonstrated that thyrotropin reference limits vary with ethnicity, with Black individuals having significantly lower TSH across the distribution, including the 2.5th, median, and 97.5th centiles, compared to White populations, indicating a leftward shift of the TSH distribution curve (19). These findings support the concept that population-specific factors, including ethnic and genetic determinants, contribute to variation in thyroid function. In addition, differences may also arise from the nature of the study population itself. Thyroid function tests obtained during hospital visits may be influenced by underlying illness or physiological stress, leading to transient alterations in TSH due to non-thyroidal illness effects. Consequently, hospital-based populations may not accurately reflect baseline thyroid function in otherwise healthy individuals and may introduce bias in the estimation of reference intervals. In contrast, community-based cohorts, such as in the present study, are more likely to provide physiologically representative estimates of thyroid function reference intervals.

In the current study, TSH levels demonstrated a dynamic trend from pre-pregnancy to the second trimester. The mean TSH levels showed a significant decline from 2.3 µIU/mL pre-pregnancy to 1.6 µIU/mL in the first trimester (P < 0.01), followed by an increase to 2.0µIU/mL in the second trimester (P = 0.02). Similarly, the 97.5^th^ percentile for TSH decreased from 4.7 µIU/mL pre-pregnancy to 3.6 µIU/mL in the first trimester, before rising slightly to 3.9 µIU/mL in the second trimester. The significant decline in TSH levels during the first trimester, followed by a modest rise in the second trimester, aligns with the well-established influence of hCG.

Current study also found that higher pre-pregnancy abdominal circumference is associated with higher first-trimester TSH levels. This suggests a link between increased baseline abdominal size and elevated TSH in early pregnancy. Thyroid hormones interact with adipose tissue, where reduced expression of TSH and thyroid hormone receptors in obese individuals leads to compensatory increases in TSH and free T3. Leptin also plays a role in regulating TSH secretion (20). However, no correlation was found between pre-pregnancy abdominal circumference and TSH levels in the second trimester.

Participants with the highest fall in TSH during the first trimester (upper 10th centile) had significantly higher pre-pregnancy TSH levels (4.5 ± 0.9 µIU/mL) compared to those with lower TSH declines (2.2 ± 0.9 µIU/mL, *p* < 0.05). This suggests a trend toward TSH normalization in the first trimester, particularly in individuals with higher pre-pregnancy TSH levels.

### Strengths

This study is among the first in India to establish trimester-specific TSH reference ranges using a community-based cohort, enhancing the generalizability of the findings to the broader population. By assessing thyroid status before conception and prospectively following participants through pregnancy, it provides valuable insights into physiological changes in thyroid function. Furthermore, the analysis of baseline clinical and laboratory parameters enabled identification of factors influencing thyroid function in pregnant women.

### Limitations of the study

The study did not include follow-up assessments in the late third trimester due to logistical constraints. In addition, urinary iodine concentration data were not available, which limited the ability to assess iodine status as a contributing factor to thyroid function variations.

## Conclusion

This community-based study in Thiruvananthapuram, Kerala, establishes the trimester-specific reference intervals for TSH and total T4. The 2.5th - 97.5th percentiles reference ranges in the first trimester for TSH and Total T4 were 0.10 - 3.9 µIU/mL and 5.2 - 17.0 µg/dl respectively, and in the second trimester, they were 0.7 - 4.6 µIU/mL and 7.6 µg/dl - 14.7 µg/dl respectively. Prepregnancy abdominal circumference has a positive correlation with first trimester TSH.

## Data Availability

The original contributions presented in the study are included in the article/supplementary material. Further inquiries can be directed to the corresponding author.
